# Urea cycle disorders and indications for liver transplantation

**DOI:** 10.3389/fped.2023.1103757

**Published:** 2023-03-03

**Authors:** Marta García Vega, José D. Andrade, Ana Morais, Esteban Frauca, Gema Muñoz Bartolo, María D. Lledín, Ana Bergua, Loreto Hierro

**Affiliations:** ^1^ Department of Pediatric Hepatology & Liver Transplant, Hospital Universitario La Paz & IdiPAZ, ERN Rare-Liver, ERN Trasplant Child, Madrid, Spain; ^2^ Department of Pediatric Nutrition and Metabolic Diseases, Hospital Universitario La Paz, Madrid, Spain

**Keywords:** liver transplant, inborn errors of metabolism, hyperammonemia, urea cycle disorders, hepatology

## Abstract

Urea cycle disorders (UCD) are inborn errors of metabolism caused by deficiency of enzymes required to convert nitrogen from ammonia into urea. Current paradigms of treatment focus on dietary manipulations, ammonia scavenger drugs, and liver transplantation. The aim of this study was to describe the characteristics and indication of liver transplantation in UCD in a tertiary hospital. We performed a retrospective study of children with UCD seen in the period 2000–2021. Data was collected on clinical onset, hyperammonemia severity, evolution and liver transplantation. There were 33 patients in the study period, whose diagnosis were: ornithine transcarbamylase (OTC, *n* = 20, 10 females), argininosuccinate synthetase (ASS, *n* = 6), carbamylphosphate synthetase 1 (CPS1, *n* = 4), argininosuccinate lyase (ASL, *n* = 2) and N-acetylglutamate synthetase (NAGS, *n* = 1) deficiency. Thirty one were detected because of clinical symptoms (45% with neonatal onset). The other 2 were diagnosed being presymptomatic, by neonatal/family screening. Neonatal forms (*n* = 14) were more severe, all of them presented during the first week of life as severe hyperammonemia (mean peak 1,152 µmol/L). Seven patients died (6 at debut) and all survivors received transplantation. There was no mortality among the late forms. Of the 27 patients who did not die in the neonatal period, 16 (59%) received liver transplantationwith 100% survival, normal protein tolerance and usual need of citrulline supplementation. The transplant's metabolic success was accompanied by neurologic sequelae in 69%, but there was no progression of brain damage. Decision of continuous medical treatment in 11 patients appeared to be related with preserved neurodevelopment and fewer metabolic crises.

## Introduction

Urea cycle disorders (UCD) are rare metabolic inherited disorders affecting the metabolism of nitrogen and endogenous synthesis of arginine. Disruption of the urea cycle caused by deficiencies of one of six enzymes and two transporters required for ammonia detoxification and urea synthesis, results in hyperammonemia, which can lead to cytotoxic brain edema and may result in death. Severe cases present in infancy with life-threatening metabolic decompensation, usually characterized by lethargy that progresses to coma, seizures, and multi-organ system failure. In those who recover from acute hyperammonemia, intellectual and developmental disabilities are common (1–3). Early diagnosis and treatment are of the utmost importance.

The prevalence of UCD vary between 1:35.000 and 1:69.000 (3–5). Although it could be higher, considering that not all the cases are detected by newborn screening.

Acute hyperammonemia requires emergency treatment with promotion of anabolism, protein restriction diet, ammonia-lowering drugs (nitrogen scavengers) and often even extracorporeal detoxification, especially in the neonatal period (1–4, 6). Long-term management aims to achieve metabolic stability and adequate growth and neurological development with restriction of natural protein intake, nitrogen scavengers and supplementation of arginine and/or citrulline (2, 3, 6). In the event of intercurrent illness or prolonged fasting, a carbohydrate emergency regimen becomes vital in preventing acute metabolic decompensation.

Despite breakthroughs in medical and dietary therapy, long-term clinical outcomes of affected individuals are often poor. It is unclear whether this is solely due to the first neurological insult or whether recurring metabolic decompensations and subclinical chronic hyperammonemia play a role. Neonatal screening in the most recent times allows early diagnosis with less severe hyperammonemia and less early cerebral damage. However, children continue to develop decompensations in the follow up, in similar frequency and severity as seen in the pre-screening period (7).

Liver transplantation (LT) emerged as a promising treatment that “cures” urea cycle abnormalities due to its ability to repair the metabolic deficiency and eliminate the risk of hyperammonemia. However, LT is a complicated surgical procedure, which carries risk of mortality and morbidity and requires a life-long regimen of immunosuppression (1–6).

The balance of risk of liver transplantation is particularly difficult in mild-moderately affected patients. This ambiguity can make treatment decisions very difficult for the families.

The aim of this study was to provide information on decisions, results, and clinical and neurological outcome in children with UCD who underwent a liver transplantation as well as the long-term follow up of the children with medical and dietary management.

## Methods

### Study design

We conducted a retrospective study with all patients under 18 years of age diagnosed with UCD who were under follow-up in the period 1/1/2010 to 31/12/2021. The diagnosis of UCD was based on clinical, biochemical and molecular data. Data was collected on clinical presentation, severity of hyperammonemia, liver function impairment, biochemical status, dietary and pharmacology management, and liver transplantation peri-and postoperative outcomes.

Parameters examined included age at time of diagnosis, age at transplantation, graft characteristics and survival, postoperative complications, and gross neurocognitive outcomes.

### Nutritional management

All patients were on protein-restricted diet and drugs aimed to decrease ammonium. Those who underwent LT had drug treatment switched from oral to intravenous (IV) and preoperative fasting was controlled by intravenous 10% glucose with appropriate electrolytes to ensure anabolism. After LT treatment consisted of unrestricted diet with caloric and protein intake according to their age and sex following the recommendations of the World Health Organization (WHO) and surveillance of citrulline levels to guide supplementation.

Patients evaluation and follow-up were made by a multidisciplinary team including pediatric hepatologists, metabolic diseases experts, surgeons, and nutritionists. Neurological and neurocognitive descriptions are based on the clinical evaluation notes from patients' pediatric neurologist. Decision whether to transplant or not was always made with the patient and family consensus.

### Statistical analysis

SPSS (IBM SPSS Statistics 24.0, IBM Corp., Armonk, New York) was used for descriptive statistics. A *p*-value of 0.05 or less, was understood to indicate statistical significance.

### Ethical approval

The procedures were carried out within the usual clinical practice and review was approved by the Clinical Research Ethics Committee of La Paz University Hospital.

## Results

### Patient characteristics

A total of 33 patients were diagnosed with a UCD at La Paz University Hospital between January 2000 and December 2021 with the following diagnosis: 20 ornithine transcarbamylase deficiency (OTC), 6 argininosuccinate synthetase deficiency (ASD), 4 carbamylphosphate synthetase 1 deficiency (CPS1), 2 argininosuccinate lyase deficiency (ASL) and 1 N-acetylglutamate synthetase deficiency (NAGS).

Thirty-one patients were diagnosed based on clinical symptoms (14 cases with a neonatal onset). Two were diagnosed by newborn screening or family history. Forty-eight percent of the patients (16 cases) were male and 52% (17) female. From the 20 cases with OTC, 50% (10) were female.

All the patients presented with hyperammonemia with altered mental status. A complete blood and urine workup was performed including liver function and metabolic study with amino acids and organic acids (Table 1). The type of UCD was identified initially suspected by the results of the blood amino acid analysis and the urine organic acids analysis. However, genetic test was performed in all patients to confirm diagnosis.

**Table 1:** Patients transplanted and not-transplanted by UCD-type and characteristics.

	Total	Transplanted	Not Transplanted
Sex			
Male	16	7	9
Female	17	9	8
Type of UCD			
OTC	20	11	9
OTC Male	10	3	7
OTC Female	10	8	2
ASS	6	2	4
CPS1	4	2	2
ASL	2	1	1
NAGS	1	0	1
Time at diagnosis			
Neonatal onset	14	7	7
Late onset	17	7	10
Biochemical analysis at diagnosis			
Mean Ammonia (µmol/L)		502	933
Mean AST (UI/L)		1,362	379
Mean ALT (UI/L)		1,213	361
Mean PT%		55	52
Mean INR		1.9	1.6
Neurological impairment			
Normal	13	5	8
Mild	10	9	1
Severe	4	2	2
Mean preventive admission		12	9

OTC, ornithine transcarbamylase deficiency; CPS1, carbamylphosphate synthetase 1 deficiency; ASS, argininosuccinate synthetase deficiency; ASL, argininosuccinate lyase deficiency; AST, Aspartate transaminase; ALT, Alanine trasnaminase; AP, Prothrombin activity.

### Disease onset

Of the 14 patients with neonatal onset, all of them were identified within the first week of life with severe hyperammonemia [mean peak 1,152 µmol/L (range 406–2484) at the first hyperammonemic episode]. All received extracorporeal detoxification by hemodiafiltration. Seven patients (50%) in this neonatal group were males with OTC.

Of the 2 cases diagnosed by newborn screening or family history, both received treatment pre-symptomatically with protein restriction diet and ammonia scavengers. There was one male with OTC who underwent a LT at 16 years of age because of frequent metabolic decompensation with normal neurological development, and one case of male citrullinemia type 1 who achieved metabolic stability with mild neurological impairment.

Seventeen patients were diagnosed after the neonatal period (late-onset). The median age of diagnosis was 2.75 years (range 0.67–17). All presented hyperammonemia [mean peak 441.4 µmol/L (range 143- 488) at the first hyperammonemic episode].

### Decision on liver transplantation

**Figure 1:**
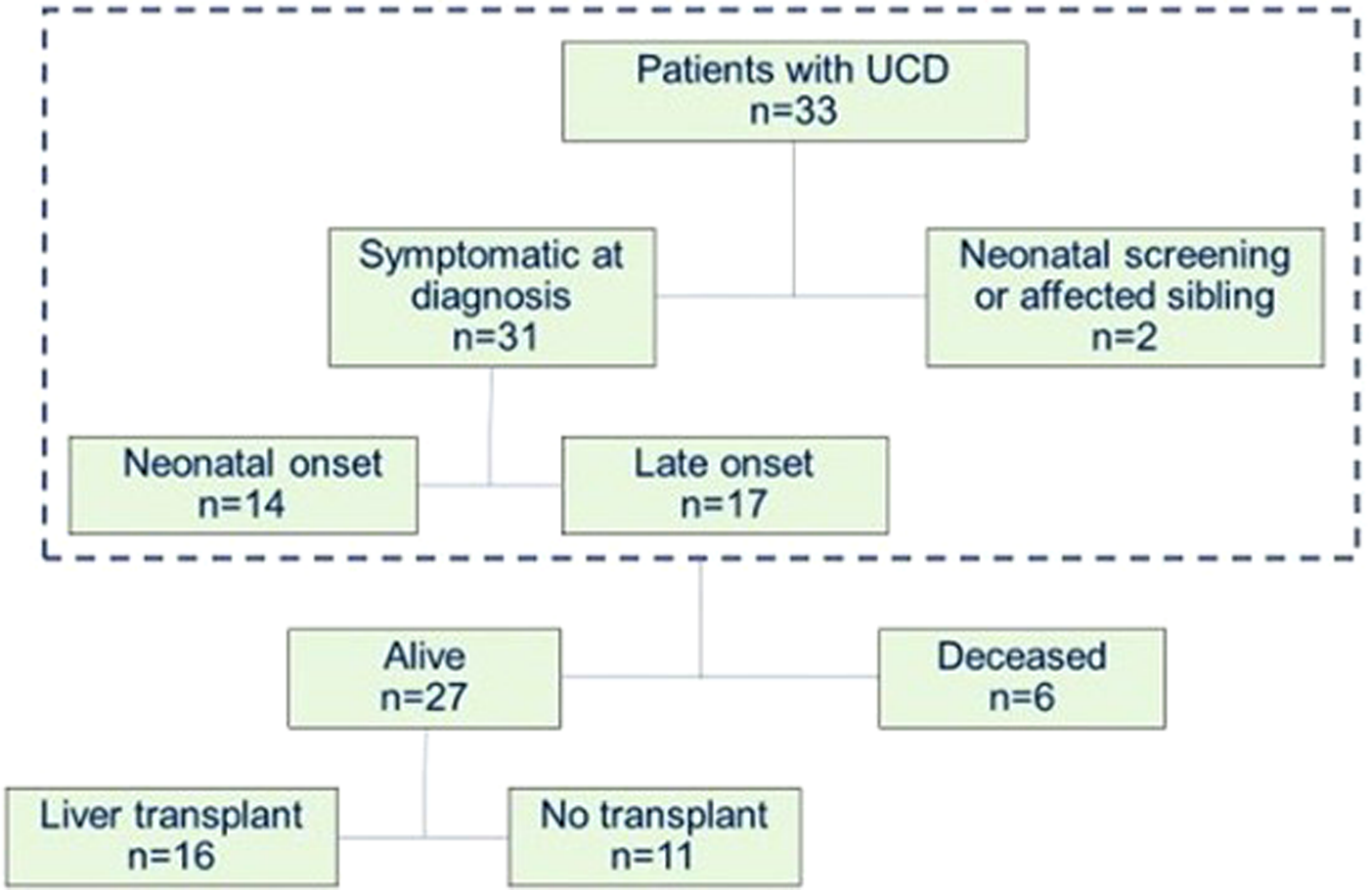
Characteristics of patients diagnosed with a UCD at La Paz University Hospital between January 2000 and December 2021.

Of the 33 patients, 6 died, all during the neonatal period: 3 OTC (100% male), 1 NAGS, 1 CPS1, and 1 ASS. Of the 27 patients who either survived the neonatal period or had a later diagnosis, 16 (59%) underwent liver transplantation (Figure 1).

Eleven patients did not undergo a LT. The reasons for not transplanting were the presence of very severe neurological sequelae in 2 patients, adequate metabolic stability in 8 cases (7 with normal neurodevelopment) and loss of data due to change of health center in 1 case. The median follow-up among those who did not receive transplantation was 7.7 years (range 0.42–29).

Sixteen patients underwent LT. The most frequent defect was OTC (11 cases, of whom 8 were female), followed by CPS1 and ASS (2 cases each) and 1 case of ASL (Table 1). Of the 16 transplant recipients, 8 were neonatal onset forms. 100% of the patients with neonatal onset forms who survived were transplanted. Of 490 pediatric liver transplants that have been performed in La Paz Hospital between 2000 and 2021, 16 were for UCD.

The median age at transplantation was 3.2 years (range 0.8–16.9), with a mean time from diagnosis to transplant of 21 months [0–203 months]. Nine were females (56%) and 7 males (44%). The median follow-up among those who received transplantation was 8.43 years (range 1.72–18.63).

The decision of transplantation was taken to prevent onset or progression of neurological damage in 15 patients and because of the presence of acute liver failure in one. The decision was made based on frequent hospitalizations despite standard medical therapy, neurological status and liver dysfunction (Table 1).

Of the 16 LT, 8 (50%) received the graft from a living donor. Because of X-linked inheritance, mothers were excluded as potential donors in OTC patients. Three patients received whole liver grafts, whereas 13 patients received cadaveric reduced-sized grafts.

### Transplantation outcomes

**Figure 2:**
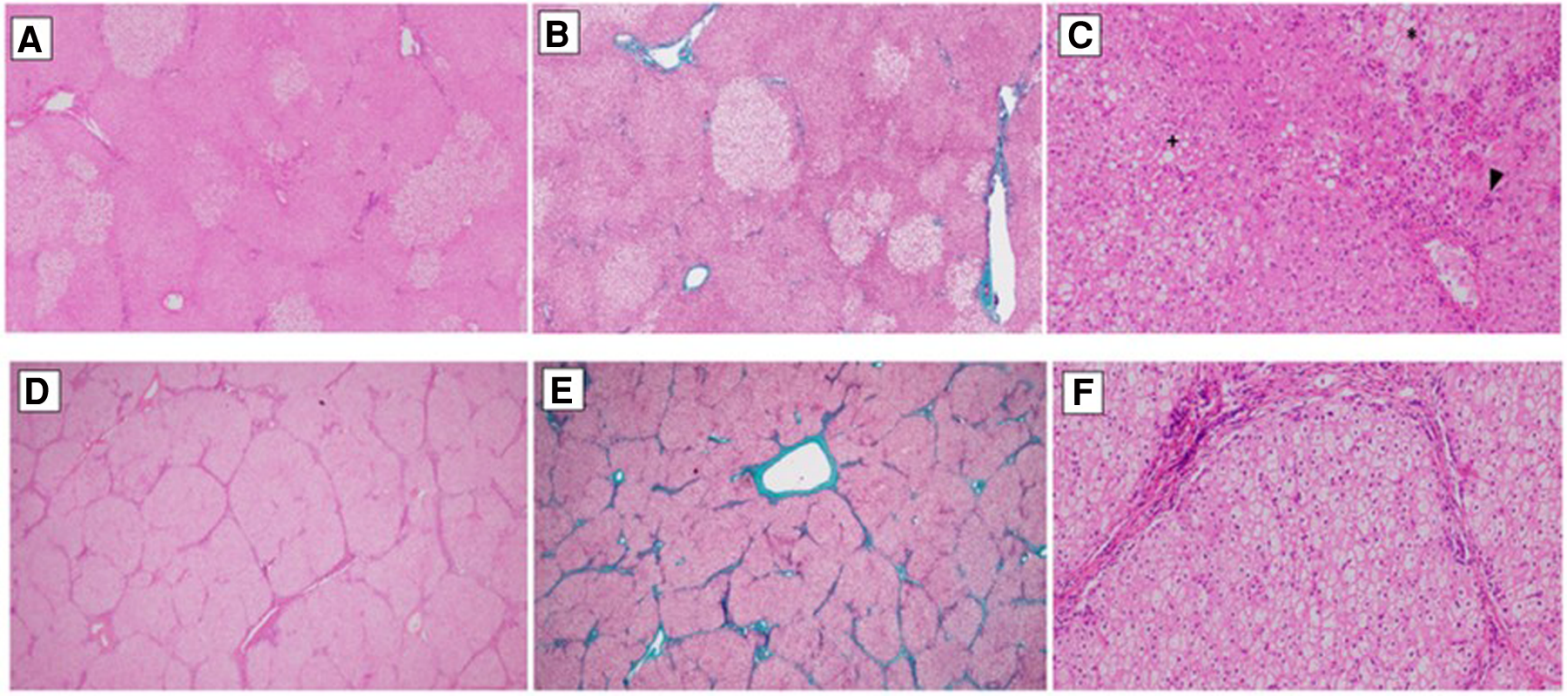
Pictures (**A–C**) show the liver explant histology of a patient diagnosed with OTC (ornithine transcarbamylase deficiency). (**A**) Preserves hepatic architecture. Patchy clear hepatocytes with no zonal distribution. Haematoxylin&Eosin, 20×. (**B**) There is no significant fibrosis. Masson's Trichrome, 20×. (**C**) Hydropic/glycogenated hepatocytes (*) admixed with macrovesicular steatotic patches (+). Around the central veins, sinusoidal aggregated neutrophils can be seen (surgical hepatitis, arrowhead). Haematoxylin&Eosin, 20×. Pictures (**D–F**) belong to a patient diagnosed with ASL (argininosuccinate lyase deficiency). (**D**) Micronodular liver architecture with both complete and incomplete septae. Haematoxylin&Eosin, 20×. (**E**) Both portal to portal and portal to central septae can be seen. Masson's Trichrome, 20×. (**F**) Hepatocytes are uniformly swollen, wih clear cytoplasm and prominent cell membrane. Masson's Trichrome, 20×.

Liver explant histology showed abnormalities in 9 of 16 explants. Eight native livers (6 OTCD and 2 CPS1) showed portal fibrosis (Metavir F1 -F2) and one ASL showed cirrhosis (F4) (Figure 2).

Post-LT, evolution was as follows:

Overall patient survival was 100% with graft survival of 94%. Complications occurred in 10 patients (62%). These complications included: 3 cases of post-operative gastrointestinal bleeding, 1 case of diaphragmatic paresia, 2 cases of acute graft rejection (both resolved with steroids), 2 cases of mild portal stenosis without significant repercussion not needing therapy, and, 1 case of refractory biliary stenosis and cholangitis that required delayed retransplantation.

Five patients required admissions for cholangitis or biliary pathology and 4 were admitted for a percutaneous transparietohepatic cholangiography procedures.

### Neurocognitive and nutrition outcomes

The neurological and neurocognitive outcomes were based on the clinical evaluation notes from patients' pediatric neurologist. Severe neurological impairment was considered in those patients diagnosed with epilepsy and cerebral palsy categorized with Gross Motor Function Classification System (GMFCS) level 5. Mild neurological impairment was described in those with motor, speech or global delay. Normal neurodevelopment included normal motor and intellectual status.

During the post-LT follow-up, neurodevelopment was found to be normal in 5 patients, with mild delay in 9 (4 OTCD, 2 CPS1D, 2 ASS and 1 ASL) and severe delay in 2 patients with OTC, both males with neonatal onset. This neurological impairment was already present prior to transplantation. The most common neurological sequelae were motor impairment found in 5 cases, followed by speech and global delay with 2 cases each, all of them categorized as mild. The severe cases were one cognitive delay and one epilepsy.

**Figure 3:**
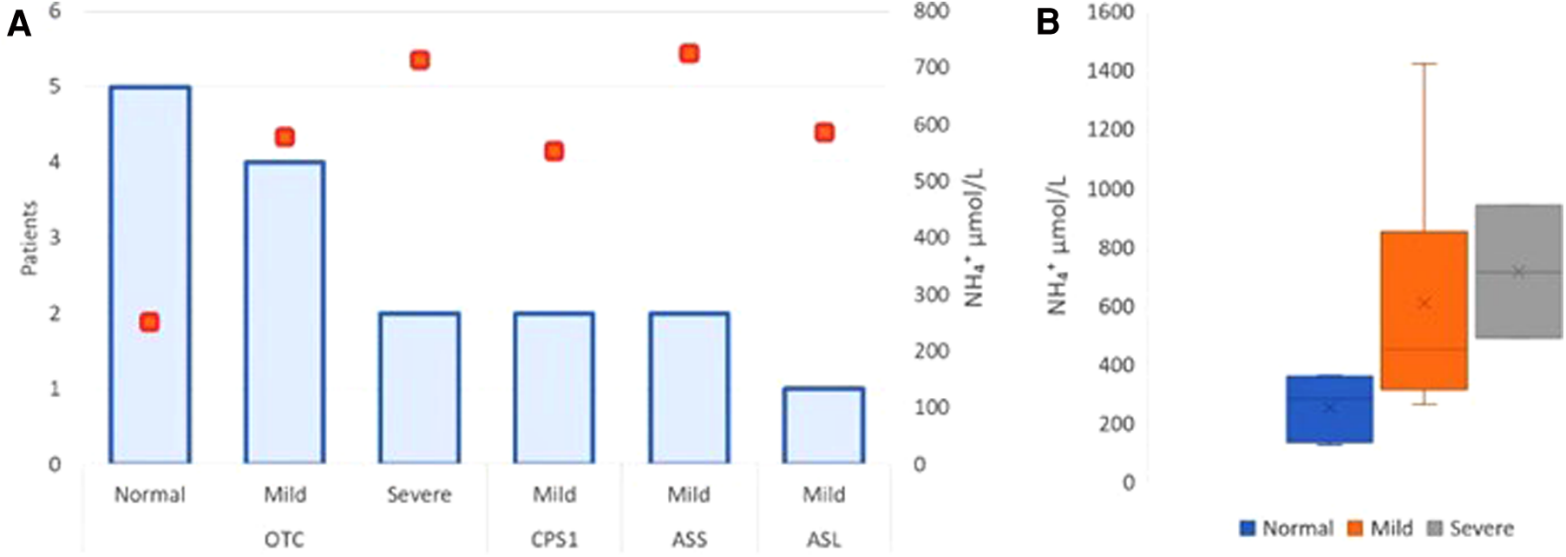
Neurodevelopmental outcomes—correlation with mean ammonium level and UCD type. Severe neurological impairment was considered in those patients diagnosed with epilepsy and cerebral palsy categorized with Gross Motor Function Classification System (GMFCS) level 5. Mild neurological impairment was described in those with motor, speech or global delay. Normal neurodevelopment included normal motor and intellectual status. The correlation with UCD type, neurological outcome, and mean ammonium level at diagnosis showed that the maximum ammonium level at onset was higher (>500 µmol/L) in those with neurological damage including mild and severe. Orange boxes show the peak of ammonium level in neurological outcome related with the UCD type. (**A**) Schematic box plots for neurological outcome show the distribution of the ammonium values within each group. The x mark shows mean on each category with its respective range: Normal 251,6 (123–361); Mild 606 (312–1420); Severe 713.5 (488–939) µmol/L) (**B**).

The correlation with UCD type, neurological outcome, and mean ammonium level at diagnosis showed that the maximum ammonium level at onset was higher (>500 µmol/L) in those with neurological damage including mild and severe (Figure 3A). However, the value was not significantly different comparing mild and severe cases (mild 606 vs. severe 713.5 µmol/L; *p* > 0.05) (Figure 3B).

Low-protein diet was no longer required in any patient after LT. At follow-up, there have been no episodes of hyperammonemia or metabolic crises in any patient after transplantation.

The mean citrulline value significantly increased post-LT (3.7 vs. 16.6 µmol/L; *p* = 0.03). Post-transplantation 9 maintained citrulline supplementation (7 OTC and 2 CPS1) because they had plasma citrulline levels persistently below reference range for age (11–45 µmol/L).

A comparison of features between non transplanted and transplanted patients showed: The peak of ammonium levels at diagnosis showed no significant differences between transplant recipients (pre-transplant) and non-transplant recipients (mean 502,25 [123–1,420] vs. 932,88 µmol/L [118–2,484]; *p* = 0.5).The mean number of preventive admissions for decompensation was 12 in those who later underwent LT vs. 9 in non-transplanted patients (*p* = 0.02), even though the observation period was much longer in the group of non transplanted children There were no significant differences in daily protein intake between both groups, transplant recipients (pre-transplant) and non-transplant recipients (mean protein intake 0.95 vs. 1.00 g/kg/day; *p* = 0.80). Neurodevelopment has already been affected by disease in 11/16 LT patients compared to 4/11 non-LT patients (*p* = 0.9).

## Discussion

We present here our experience over 21 years with patients with urea cycle defects and liver transplant decision and outcomes in these patients. Of 490 pediatric liver transplants that have been performed in La Paz Hospital between 2000 and 2021, 16 were for UCD (3.3%). The proportion of pediatric liver transplants performed for UCD increased from 0.4% in 2000–2010 (1/229 LT) to 5.7% from 2011 to 2021 (15/261). Similar to our center, LT registries document the shift to increasing numbers of children undergoing LT for UCDs in more recent times. According to the USA LT registry there were 131 UCDs in 1997–2006 and 219 in 2007–2017 (8).

Pediatric LT now has 5- and 10-year survival rates that are significantly above 90%. As the morbidity and mortality risks of LT have gradually declined, and postoperative outcomes have improved, the indications have turned from “life-saving” to “life-improving” (2–6). LT has become a well-established therapy option for urea cycle abnormalities, with positive results and improvements in neurocognitive development. However, in general it will not revert established neurological damage (1–6, 9–10).

Post-transplant survival rates are particularly high in patients with metabolic disorders as shown in the USA and European Registries and hospital series (8, 11–16). The results from our center support these data.

In UCD observed in our center in the last 21 years, the neonatal onset form was more severe, and all survivors received transplantation. In OTC, only males showed neonatal mortality. Among females, the proportion of transplant recipients was 73%. In the late forms, transplantation was performed in 59% of the patients. The mean age at the time of transplantation was 3.2 years, similar to some series described in literature (17).

The decision for transplantation was made on an individual basis by the medical team. However, it usually was in favour of transplantation. Preventing neurological damage or its progression, and metabolic stability without complications were the factors influencing the decision on transplantation.

As a summary of the analysis, families of children who had avoided cerebral damage and had well controlled episodes of metabolic risk/decompensations were the ones who decided not to transplant. Neonatal-onset UCD have significantly worse neurodevelopmental prognoses than late-onset UCD, which was consistent with our study. The age of the patients at the diagnosis and at transplantation may further influence the neurological outcome and should always be considered (1).

Regarding the ammonium level, the higher the blood ammonia concentration, the greater the brain damage. Patients with ≥360 µmol/L at onset are likely to have poor long-term outcomes even if they have received the current established treatment for UCD (2, 18). In our study the mean ammonium level at diagnosis was 1,152 µmol/L in the neonatal-onset group and 441.4 µmol/L in the late-onset group. Therefore, all the neonatal-onset patients underwent LT.

Patients described without developmental delay before transplantation have maintained their cognitive abilities at follow-up. Those who had been diagnosed with some degree of developmental delay before transplantation remained stable after transplantation.

In our study we analyzed the histological alterations found in the explanted native livers. A variety of non-specific features such as fatty change, mild inflammation, cholestasis, and focal necrosis were seen in the UCD patients. However, one case of our series, late onset OTC, presented fibrosis, focal inflammation, and pallor of the hepatocytes due to accumulation of glycogen particles, resembling to a glycogen storage disorder (19, 20). We found no particular association between the native liver histopathology and patient clinical status except for one patient with ASL who developed progressive fibrosis and cirrhosis in the histopathological study.

To maintain metabolic stability all the patients were under protein restriction diet (1, 4). However, the amount of protein tolerance did not seem to be determinant in the decision of transplant. Following transplantation, there were no further episodes of hyperammonemia, and medical and dietary therapies were terminated.

After LT, the lack of citrulline recycling in OTC and CPS1 and the *de novo* arginine synthesis persists in all UCDs except ARG1 (1), suggesting the importance of the extrahepatic function of the urea cycle.

Because citrulline is also produced outside the liver (intestinal citrulline), plasma citrulline may continue to be low (18, 21). Therefore, 9 of our patients (7 OTC and 2 CPS1) required long-term citrulline supplementation after transplantation.

Liver transplantation seems to be the ideal solution for urea cycle defects in order to prevent the onset or progression of cognitive impairment. Nutritional and developmental catch-up is most complete in those undergoing early transplantation (22, 23). Moreover, liver transplantation has evolved over the past decades to the stage in which it has become a very successful technique. We advocate for early evaluation and inclusion on waiting liver transplant list.

Future research is required in order to offer more detailed guidance and identify potential risk groups for morbidity and mortality among patients with UCD candidates for liver transplantation. This demands a significant participation and effort of transplantation institutions, as well as a prospective follow-up.

For now, it remains unanswered which patients need to be transplanted. However, recommendation guidelines have been published in order to evaluate and provide better healthcare and information to these patients and their families. If patients are considered for transplantation, it is crucial to recognize that pre- and perioperative morbidity and nutritional status are related with the prognosis after transplantation.

## Data Availability

The raw data supporting the conclusions of this article will be made available by the authors, without undue reservation.
